# Risk Assessment of Heavy Metals in Surface Sediments from the Yanghe River, China

**DOI:** 10.3390/ijerph111212441

**Published:** 2014-11-28

**Authors:** Jing Li

**Affiliations:** Key Laboratory of Ecosystem Network Observation and Modeling, Institute of Geographic Sciences and Natural Resources Research, Chinese Academy of Sciences, Beijing 100101, China; E-Mail: jingli@igsnrr.ac.cn; Tel./Fax: +86-10-64-889-300

**Keywords:** emergency pollution event, Index of geoaccumulation, community bureau of reference (BCR), sediment quality guidelines, risk index

## Abstract

The magnitude and ecological relevance of metal pollution from the upstream of water sources after emergency pollution events was investigated by applying a set of complementary sediment quality assessment methods: (1) geochemical assessment based on background value (the geoaccumulation index); (2) comparisons with sediment quality guidelines (SQGs); (3) an evaluation of the combined pollution according to the risk index (RI); and (4) investigation of the chemical patterns of target heavy metals (Cd, Zn, Cr, Pb, Ni). The geoaccumulation indices (*I_geo_*) suggested that the magnitude of heavy metal pollution of the sediment of Yanghe River decreased in the order of Cd > Zn > Pb > Cr > Ni. Risk analysis also suggested that Cd and Zn concentrations were sufficiently elevated as to cause adverse biological effects in this study area. According to the RI values, 27% of total sampling sites showed considerable ecological risk for the water body, and 53% of total sampling sites showed very high ecological risk for the waterbody. Sediment-bound Cd was found to be predominantly associated with the exchangeable phase of the sediment (25%–68%), while Cr, Ni, Zn and Pb showed the strongest association with the residual fractions (60%–92%, 53%–67%, 24%–85% and 35%–67%, respectively).

## 1. Introduction

In the past few decades, heavy metals accumulation in the environment has been attracting increasing attention from both researchers and policymakers because of their toxicity, persistence in the environment and subsequent accumulation in aquatic habitats [1,2,3,4,5]. Sediments play a major role in determining the pollution patterns of aquatic systems. They act as both carriers and sinks for contaminants, reflecting the history of pollution, and providing a record of catchment inputs into aquatic ecosystems [6,7,8,9].

The Guanting Reservoir is situated in the northwest part of Beijing [10]. Historically, Guanting Reservoir has been used as one of the two sources of water for agriculture, industry, and municipalities in Beijing [11,12]. Since the 1970s, the Yanghe River, upstream of Guanting Reservoir, has undergone significant agricultural and industrial development and consequently water in Guanting Reservoir has become contaminated. Two well-known pollution events took place in 1972 and 1989. In 2009, the third pollution event happened along the Yanghe River, and the total damaged farmlands were approximately 12–18 km^2^ [13,14]. Because of this, since 1997, the Guanting Reservoir is no longer been used as a source of drinking water for Beijing. Recently, the government has been reconsidering using the Guanting Reservoir for drinking water supply, because of severe water shortages in Beijing. However, another emergency pollution event along the Yanghe River took place in 2009, causing fish in the aquatic ecosystem to die while residents who drank the water became sick. Our sampling plan and investigation was carried out shortly after the emergency pollution event. The data provided in this study is considered to be very important for reservoir remediation, especially since the Guanting Reservoir will serve as one of the main drinking water sources for Beijing in the foreseeable future.

On the basis of earlier researches [15,16,17,18], a comprehensive and reliable scheme of the preliminary risk assessment of metal contamination in surface sediments should contain: (1) comparisons with background values; (2) an evaluation of combined pollution; (3) comparisons of concentrations with the values of sediment quality guidelines; and (4) an investigation on partitioning the carrier operationally defined phases of metals.

The sediment quality guidelines (SQGs) have been used to identify “contaminants of concern” in aquatic ecosystems and to rank “areas of concern” on a regional or national basis, and have also been used in numerous other applications, including the design of monitoring programs, interpreting historical data, conducting remedial investigations and ecological risk assessments and so on [19,20]. SQGs are very important for protection of benthic organisms in freshwater ecosystems and can be used to assess sediment ecosystem health [7]. Many different SQGs have been developed [19,21]. We chose to adopt two levels of sediment quality guidelines in this study: the TEL and PEL. These two assessment values, delineated three ranges of chemical concentrations that were rarely (minimal effect range; concentrations equal to and below the TEL), occasionally (possible effect range; concentrations above the TEL, but below the PEL), and frequently (probable effect range; concentrations equal to and above the PEL) associated with adverse biological effects [22].

All of mentioned approaches focus on ecological risk assessment of a single metal. However, naturally occurring heavy metal pollution is in the form of combined pollution. The potential ecological risk index developed by Hakanson is based on the assumption that the sensitivity of the aquatic system depends on its productivity. This methodology was a very useful and comprehensive methodology which can be used to evaluate the combined pollution risk of an aquatic system through a toxic-response factor for a given substance [23].

Due to chemical and geological conditions, heavy metals in sediments can exist in different forms: soluble, exchangeable, bound to organic matter, occluded in Mn and/or Fe oxides, as a component of carbonates, phosphates, sulphurs, or other secondary minerals, or bound to silicates (residual) [9]. Thus, the carrier operationally defined phases analysis of heavy metals might provide much useful information regarding the chemical nature or potential mobility and bioavailability of a particular element, which consequently can offer a more realistic estimate of actual environmental impact [24].

The objectives of the research paper were to: (1) determine concentrations of the metals cadmium (Cd), nickel (Ni), lead (Pb), chromium (Cr) and zinc (Zn) in surface sediments of the Yanghe River; (2) to investigate the environmental risk associated with the metal contents of sediments using available SQGs; (3) to assess the combined pollution of target metals in different sampling sites; and (4) to determine the chemical carrier operationally defined phases of heavy metal (Cd, Cr, Pb, Ni and Zn) in sediment from the Yanghe River in order to evaluate their relative mobility and bioavailability.

## 2. Materials and Methods

### 2.1. Study Area

Guanting Reservoir, is located to the northwest of Beijing (E115.43º, N40.19°–E115.97°, N40.50°), which includes 98 km^2^ of water and 820 km^2^ of land. Our study area was the 106 km long Yanghe River, upstream of Guanting Reservoir, with a total catchment area of 9762 km^2^. Weather in the area is a cool temperature continental monsoon climate, with an average annual temperature between 3 °C and 9 °C and a mean annual precipitation between 370 and 480 mm. The annual accumulated temperature greater than 10 °C ranges from 2100 to 3600 °C. Land use in the region includes farms, orchards, fallow lands and lots of industrial land. There were more than 300 industrial and mining enterprises, producing about 7.8 × 10^7^ t of wastewater annually. Most of this waste water was discharged into the river and reservoir without any treatment.

### 2.2. Sediment Sampling

**Figure 1:**
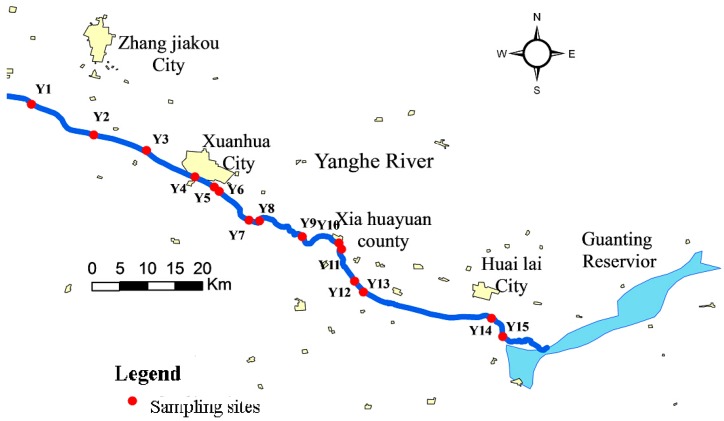
Sampling sites along the Yanghe River.

In August of 2009, 15 sediment samples were collected along the upstream of water source. The sampling sites were located using a global positioning system. The distribution of the sampling points is shown in Figure 1. At each sampling point, approximately the top 5 cm of sediments were collected using a self-made sediment cores sampler. All samples were sealed in clean polyethylene bags and put in a cooled box on site. The cooled samples were brought back to the lab to be freeze-dried until required.

### 2.3. Metal Analysis

The samples were freeze dried and then ground to a small enough size to pass through a 200-mesh sieve and digested with a mixture of concentrated HCl-HNO_3_-HF-HClO_4_ [25]. About 0.25 g of sediment was subjected to a digestion solution, and the mixture then was heated on an electric heating plate at 200 °C. After being perchloric acid smoked, the mixture was heated for another 3 min then cooled to room temperature. After cooling, the residue was digested again with the digestion solution at 200 °C to dryness. The detection limits were 0.02 mg/kg for Cd, 0.2 mg/kg for Cr, 1 mg/kg for Ni, Pb and Zn. Samples were carefully handled to avoid introduction or loss of trace elements during preparation and analysis. All materials used during analytical determinations were kept in Teflon or other metal-free containers.

Chemical carrier operationally defined phases of traces in the sediment samples was performed based on the Bureau of Reference (BCR) sequential extraction procedure (SEP) [26,27]. The standard reference material (BCR 701) was used to verify the accuracy of the sequential extraction method. The recovery rates for heavy metals ranged from 86% (for Zn in the exchangeable phase) to 116% (for Cr in the residual fraction). This gave partitioning in four chemical phases: (1) exchangeable; (2) Fe/Mn oxides; (3) organic matter/sulfides; (4) residual fraction. All supernatants were decanted into acid washed polyethylene containers and refrigerated prior to analysis. Between each phase of the SEP, samples were washed using 10 mL Milli-Q water. The extraction was performed for 0.5 g of dried sediment samples in 50 mL polypropylene centrifuge tubes. The detailed procedure used in this experiment is given in Table 1.

**Table 1:** Sequential extraction scheme for heavy metal fractionation in sediments.

Step	Phases	Procedure
1	Exchangeable fraction	0.11 M ammonium acetate, 20 mL, room temperature, shake for 16 h
2	Fe/Mn oxides	0.5 M hydroxylammonium chloride (pH 2), 20 mL, room temperature, shake for 16 h
3	Organic matter/sulfides	8.8 M hydrogen peroxide (pH of 2–3), 5 mL, room temperature, 1 h, followed 85 °C, 1 h, occasionally shake; add 5 mL 8.8 M hydrogen peroxide (pH of 2–3), 85 °C, 1 h; add 1 M ammonium acetate (pH = 2), 25 mL, room temperature, shake for 16 h
4	Residual fraction	A mixture of concentrated HCl-HNO_3_-HF-HClO_4_

Concentrations of the metals Cd, Ni, Pb, Cr and Zn in the digestion solution were determined by ICP-MS. Quality control was assured by the analysis of duplicate samples and standard reference materials. The standard reference materials (GSS-1 were used for total element analysis; percentage recoveries ranged from 94% (for Cd) to 114% (for Zn). In majority of cases the sums of the extracted fractions agree, to within 15%, with the independently determined total metal concentrations discussed above, supporting the overall accuracy of the extraction procedure. Analytical precision, expressed as relative standard deviation, was generally better than 10%.

### 2.4. Statistical Analyses

Statistical analyses were conducted by use of Microsoft Excel and SPSS 13.0 statistical software on a personal computer, and the results included maximum, minimum, mean, skew and Coefficient of Variation (CV). The distribution of concentrations was tested with the Shapiro-Wilk method to determine if they approximated the normal probability distribution.

## 3. Results and Discussion

### 3.1. Levels of Heavy Metal

The descriptive statistics for the concentrations of heavy metals in the sediments of the Yanghe River are presented in Table 2. The Shapiro-Wilk test indicated that the concentrations of all of metals followed normal distributions (S-W *p* > 0.05). The heavy metal background levels in soils of Hebei Province were used as background values. The mean concentration of Cd (3.78 mg/kg, dw) was greater than its background value (0.19 mg/kg, dw). All of sediment samples significantly exceeded the background value. The mean concentration of Zn was 2.40 × 10^2^ mg/kg, dw, which was significantly greater than the corresponding background value of 73.6 mg/kg, dw. The range of Zn (905 mg/kg, dw) between the minimum and maximum values, was greatest of the elements studied and had a coefficient of variation of 119% and skew of 1.72 which were also the greatest of the elements studied. This observation is the result of the heterogeneity of concentrations of Zn with a few locations having greater concentrations. The number of samples significantly exceeding the reference value for Cr, Pb and Ni were 9, 8 and 3, respectively.

**Table 2:** Heavy metal concentrations in Yanghe River sediments (mg/kg).

Index	Cr	Cd	Zn	Pb	Ni
Max	111	8.66	942	59.3	36.8
Min	40.7	0.64	37.1	ND	10.8
Mean	74.7	3.78	238	23.1	20.5
Coefficient of variation %	32.5	54.6	119	66.6	36.8
Skew	0.09	0.80	1.72	0.57	0.84
S-W *p*	0.34	0.63	0.09	0.63	0.23
Background value of Hebei soil **^a^**	63.8	0.19	73.6	22.4	25.4
Number exceeding reference values **^b^**	9	15	9	8	3

Notes: ND, not detectable; **^a^**: Xu, *et al.* [28]; **^b^**: The number whose value is significantly greater than the corresponding reference value.

The greatest concentrations of Cd, Pb and Zn occurred at location Y8, while the greatest concentrations of Cr and Ni were found at sites Y12 and Y1, respectively. Coal-related industries, such as iron and steel plants, power stations and coal yards, have developed significantly along the bank of Yanghe River. For example, the Xuanhua iron and steel plant that was established 80 years ago, is located upstream of Y8 [14]. Due to the smelting of iron and steel plants, metals including Cd, Pb and Zn could be discharged into river [29,30]. Zheng *et al*. [31] studied the metal contamination in the sediments of rivers in Huludao City, and found that the Huludao Zinc Plant was the main reason for the metal pollution in the river. There were also lots of coal yards located nearby the site Y8 [28]. Coal which contains high metals concentrations could be transported to the river by rainfall. In addition, higher Cd and Ni concentration might be ascribed to the effluents of variety of industrial plants scattered along the river bank (leather factory, flour mill, oil refining plants).

**Figure 2:**
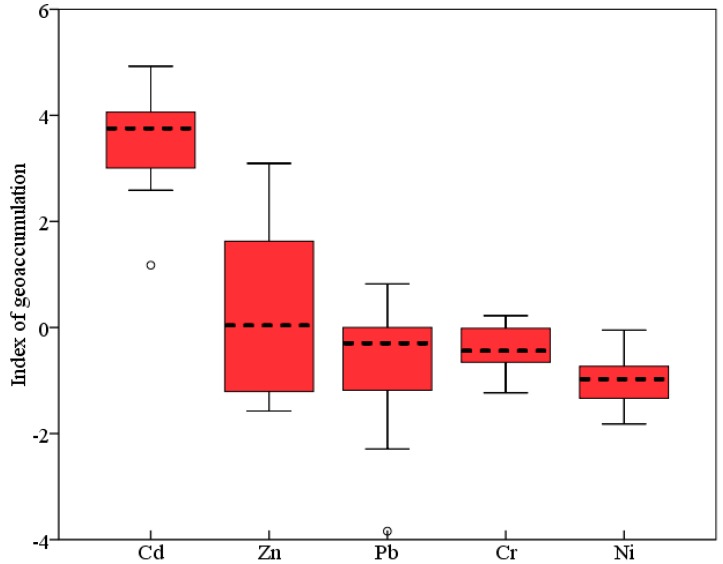
Box-and whisker plots of the index of geoaccumulation of metals in the sediment of Yanghe River.

The geoaccumulation index (*I**_geo_***) was also used to assess metal pollution in sediments of Yanghe River. The geoaccumulation index is expressed as follows:

(1) $$I_{geo}\;={\text{ log}}_{2}[\frac{C_{n}}{{\text{1.5}B}_{n}}]$$

where *C*_n_ is the measured concentration of the heavy metal (n) in the sediment, *B*_n_ is the geochemical background value in the average shale of element *n*, and 1.5 is the background matrix correction factor due to lithogenic effects. The *I*_geo_ is associated with a qualitative scale of pollution intensity, seven classes of pollution from unpolluted (*I*_geo_ ≤ 0) to extremely polluted (*I*_geo_ ≥ 5) are defined for the quality of sediments based on *I_geo_* values [32]. Following this classification the surficial sediments collected along the upstream of Guanting Reservoir can be categorized as strongly polluted with Cd (3 < mean *I*_geo_ < 4), unpolluted to moderately polluted with Zn (0 < mean *I*_geo_ < 1), and unpolluted with Cr, Pb and Ni. In conclusion, based on the *I*_geo_ classification, the magnitude of heavy metal pollution of the sediment of Yanghe River decreased in the order of Cd > Zn > Pb > Cr > Ni (Figure 2).

### 3.2. Sediment Quality Guidelines

The heavy metal concentrations were compared with the TEL and PEL values and the results presented in Table 3. The percentages of samples exceeding the TEL and PEL values for Cd were 100% and 53%, respectively. Among the investigated elements, Cd most often exceeded the TEL and PEL values. The percentages of samples exceeding the TEL and PEL values for Zn were 40% and 33%, respectively. The percentages of samples exceeding the TEL value for Pb, Ni and Cr were 13%, 67% and 93%, respectively. These three trace elements in the sediment samples fell below the PEL value in all sediment samples. However, exceedance of SQG values does not firmly guarantee the occurrence of deleterious ecological effects, unless they are also coherent with regional background levels [8,33,34]. Although 93% and 67% sediment samples had concentrations of Cr and Ni exceeding their respective TEL, which were expected to have adverse biological effects occasionally, however, 60% and 20% of sediment samples had the concentration of Cr and Ni exceeding their respective regional background levels. Therefore, contamination ranking of Cr and Ni load of sediments against the regional background levels seems to be more reliable. This result is agreement with consequences reported in the literature [8].

**Table 3:** Comparison of sediment quality guidelines with average values for the Yanghe River.

Index	Sediment Metal Concentrations (mg/kg)				
	Ni	Cr	Pb	Zn	Cd
TEL	16.0	42.0	35.0	123	0.60
PEL	43.0	160	91.0	315	3.50
Average values for the study area	20.5	74.7	23.1	238	3.78
% samples which exceeded TEL	67	93	13	40	100
% samples which exceeded PEL	0	0	0	33	53

### 3.3. Comparative Ecological Impact Assessment

To assess the effect of multiple metals pollution in the sediments from the upstream of water source, the quantitative approach developed by Hakanson was used. According to this methodology, the potential ecological risk index (RI) is defined as:

(2) $$RI=\sum_{i=1}^{n}\left(T_{i}\times \frac{C_{i}}{C_{o}}\right)$$

where *T_i_* is the toxic-response factor for a given substance (e.g., Cd = 30, Pb = Ni = 5, Cr = 2, Zn = 1); *C_i_* represents metal content in the sediments; and *C_o_* is the regional background value of heavy metals in the sediments. In this study, the heavy metal content of soils in Beijing (Table 2) was used as the regional background value. The RI value is associated with a qualitative scale of pollution intensity, samples may be classified as following: RI < 150—low ecological risk for the waterbody; 150 < RI < 300—moderate ecological risk for the waterbody; 300 < RI < 600—considerable ecological risk for the water body; RI > 600—very high ecological risk for the waterbody.

**Figure 3:**
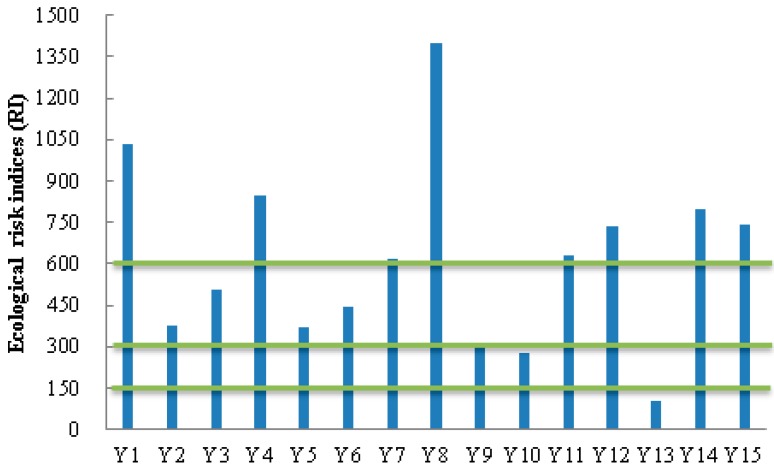
Potential ecological risk indices (RI) of heavy metals in surface sediments from the Yanghe River.

The RI of sediment samples from the upstream of Guanting Reservoir are compared in Figure 3. The RI value for the sampling site Y13 was lower than 150, suggesting that this sampling site exhibited low ecological risk from heavy metal pollution. Four sampling sites (Y2, Y3, Y5, and Y6) along the Yanghe River had RI values ranging from 300 to 600, which indicate high considerable risk from heavy metals pollution. RI values of the rest sediment samples (53% of total sampling sites) were higher than 600, which mean high ecological risk of heavy metals.

### 3.4. The Carrier Operationally Defined Phases of Heavy Metals

**Figure 4:**
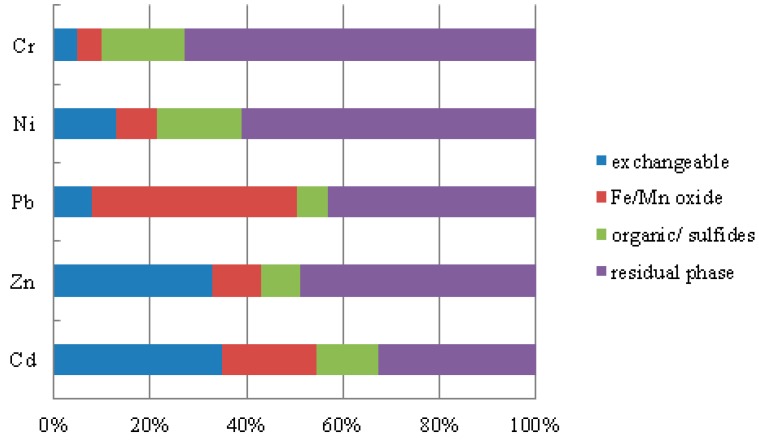
Partitioning of heavy metals in the sediments.

The carrier operationally defined phases distribution of heavy metals in sediments is of importance to investigate the metal mobility and availability. The chemical partitioning of trace metals in the sediment samples among the exchangeable, Fe/Mn oxides, sulfides/organic matter and residual fractions, is plotted in Figure 4. Each geochemical fraction is presented as the percentage of the sum of all fractions.

As Figure 4 shows, we may know that the fractionation patterns of Cr and Ni in different sediment samples were somewhat similar. Both of the sediment samples were characterized by a dominance of the residual fraction, 56%–92% for Cr and 53%–67% for Ni, respectively. The second phase is organic/sulfides with 5.0%–29% for Cr and 12%–25% for Ni, respectively. Minor contributions were found in the exchangeable phase and Fe/Mn oxides. Because the exchangeable phases are entirely negligible pollutants, even though the total concentration of Cr in the river was rather high (from 40.70 mg/kg to 111.62 mg/kg), we can conclude that Cr and Ni in the river are negligible pollutants, and mainly derived from natural processes such as weathering and soil formation.

Throughout the river stretch, lead preferentially bound to residual phase (35%–67%) and to Fe/Mn oxides (21%–56%), while the exchangeable phase and organic matter/ sulfides accounted for 3%–17% and 3%–12%, respectively. Although the average percentage of the exchangeable phase was not high (8%), with the mean value of total content of Pb (23.1 mg/kg), the Fe/Mn oxide phase could be easily released from the sediment phase into the water with changing environmental conditions. As a type of environmental hormone, Pb can affect the procreation ability of a biological system [24]. Therefore, once Pb is transferred from the sediment to the water, it poses a higher ecological risk. Therefore, the potentially Pb pollution in the river should be considered.

Zn was mainly associated the residual phase (24%–85%), followed by the exchangeable phase (5%–62%), Fe/Mn oxide (4%–22%) and organic/ sulfides (4%–17%). Among the investigated elements, the variation of Zn in different fraction of sediment samples is highest of all. High content of Zn in exchangeable phase and total extractable Zn in some sediment samples was found to be consistent with the high total concentrations of Zn in these sampling sites. Taking these two factors into consideration, the potential Zn pollution in the river should be given more attention.

The operationally defined carrier phases of Cd were: exchangeable (35%) > residual phase (33%) > Fe/Mn oxide (19%) > organic/ sulfides (13%). Low levels of Cd in the organic/sulfides can be due to the low adsorption constant and labile complex with organic matter [35]. A high content of total extractable Cd was observed in all the sediments (59%–88%), with significant amounts of Cd present in the exchangeable phase (25%–68%). Among the investigated elements, Cd was most strongly present in the exchangeable phase. Cadmium has always been ever considered as a significant metal for environmental behavior. In addition, the total concentration of Cd in the river was also very high (from 0.64 to 8.66 mg/kg). It can be concluded that potential Cd pollution in the river should be treated seriously.

Based on the analysis of carrier operationally defined phases of metals in the surface sediment along the Yanghe River, the proportion of residual fraction of Cd, Pb and Zn were lower than Cr and Ni, which means the proportion of Cd, Pb and Zn come from anthropogenic sources were higher than the other two metals [36,37]. Industrial development along the Yanghe River has been quick and intensive. There are lots of companies along the bank of the river, such as iron and steel plants, power stations and oil refining plants and so on [13]. In addition, there were also several highways across this area with heavy traffic flow in the past several years [14], so we thought the wastewater from the plants, the atmospheric deposition from energy production and metallurgical industries and the discharge of pollutants from vehicle exhaust might be the possible anthropogenic sources for metals, especially for Cd, Pb and Zn [36].

## 4. Conclusions

This study investigated the magnitude and ecological relevance of metal pollution from the upstream of Yanghe River after an emergency pollution event by applying a set of complementary sediment quality assessment methods. According to the geoaccumulation index (*I_geo_*) classification, this area has to be considered as “strongly” polluted with Cd, and “unpolluted to moderately” polluted with Zn. Comparisons with sediment quality guidelines revealed that Cd and Zn are considered as two principal traces in samples to frequently cause adverse biological effects. From our combined assessment, we judge most sampling sites to be high ecological risk areas. Investigation of the chemical pattern of elements in sediments from the river indicated that high content of total extractable Cd and Pb were observed in all sediments, Zn was mostly enriched in the residual phase and Fe/Mn oxides, and Cr and Ni were primarily present in the residual phase.

The metals of greatest concern in the sediments of the Yanghe River were found to be Cd and Zn. Almost the whole river was contaminated with either or both types of metal. We estimate that heavy metals pollution was one of major contributors to the emergency pollution event in 2009. In order to be able to again use Guanting Reservoir as one of the main drinking water sources for Beijing, remediation and conservation is required along the Yanghe River as soon as possible. This should include: (a) treatment of domestic and industrial sewage; (b) closure of mine works; (c) reduction in the overall primary industrial activities; and (d) political environmental protection policies and actions.
